# Remission of severe restless legs syndrome and periodic limb movements in sleep after bilateral excision of multiple foot neuromas: a case report

**DOI:** 10.1186/1752-1947-4-306

**Published:** 2010-09-17

**Authors:** Ludwig A Lettau, Charles J Gudas, Thomas D Kaelin

**Affiliations:** 1 Lowcountry Infectious Diseases, Charleston, SC, USA; 2 Associated Foot Specialists, Charleston, SC, USA; 3 South Carolina Sleep Medicine, Summerville, SC, USA

## Abstract

**Introduction:**

Restless legs syndrome is a sensorimotor neurological disorder characterized by an urge to move the legs in response to uncomfortable leg sensations. While asleep, 70 to 90 percent of patients with restless legs syndrome have periodic limb movements in sleep. Frequent periodic limb movements in sleep and related brain arousals as documented by polysomnography are associated with poorer quality of sleep and daytime fatigue. Restless legs syndrome in middle age is sometimes associated with neuropathic foot dysesthesias. The causes of restless legs syndrome and periodic limb movements in sleep are unknown, but the sensorimotor symptoms are hypothesized to originate in the central nervous system. We have previously determined that bilateral forefoot digital nerve impingement masses (neuromas) may be a cause of both neuropathic foot dysesthesias and the leg restlessness of restless legs syndrome. To the best of our knowledge, this case is the first report of bilateral foot neuromas as a cause of periodic limb movements in sleep.

**Case presentation:**

A 42-year-old Caucasian woman with severe restless legs syndrome and periodic limb movements in sleep and bilateral neuropathic foot dysesthesias was diagnosed as having neuromas in the second, third, and fourth metatarsal head interspaces of both feet. The third interspace neuromas represented regrowth (or 'stump') neuromas that had developed since bilateral third interspace neuroma excision five years earlier. Because intensive conservative treatments including repeated neuroma injections and various restless legs syndrome medications had failed, radical surgery was recommended. All six neuromas were excised. Leg restlessness, foot dysesthesias and subjective sleep quality improved immediately. Assessment after 18 days showed an 84 to 100 percent reduction of visual analog scale scores for specific dysesthesias and marked reductions of pre-operative scores of the Pittsburgh sleep quality index, fatigue severity scale, and the international restless legs syndrome rating scale (36 to 4). Polysomnography six weeks post-operatively showed improved sleep efficiency, a marked increase in rapid eye movement sleep, and marked reductions in hourly rates of both periodic limb movements in sleep with arousal (135.3 to 3.3) and spontaneous arousals (17.3 to 0).

**Conclusion:**

The immediate and near complete remission of symptoms, the histopathology of the excised tissues, and the marked improvement in polysomnographic parameters documented six weeks after surgery together indicate that this patient's severe restless legs syndrome and periodic limb movements in sleep was of peripheral nerve (foot neuroma) origin. Further study of foot neuromas as a source of periodic limb movements in sleep and as a cause of sleep dysfunction in patients with or without concomitant restless legs syndrome, is warranted.

## Introduction

Restless legs syndrome (RLS) is a sensorimotor neurological disorder characterized by an urge to move the legs in response to uncomfortable leg sensations [1]. While asleep, 70% to 90% of patients with RLS have periodic limb movements of sleep (PLMS). Frequent PLMS and related brain arousals as documented by polysomnography are associated with poorer quality of sleep and daytime fatigue. RLS onset in middle age (late onset RLS) is sometimes associated with neuropathic foot dysesthesias (numbness, burning and/or tingling, lancinating electric shock pains, and hypersensitivity), which are attributed to small fiber-type peripheral neuropathies [2]. We have previously reported a case series of patients with diabetes or human immunodeficiency virus (HIV) whose dysesthesias of the feet were due to bilateral Morton's neuromas rather than neuropathy [3].

A neuroma is a focal enlargement of the second, third (Morton's), or fourth digital nerve in the forefoot where each nerve stretches under the deep transverse ligament between the respective adjacent metatarsal heads [4]. Repeated nerve stress results in pathological changes of so-called entrapment neuropathy, including thickening and degenerative enlargement of the nerve and surrounding fibrous sheath into a nerve impingement mass [5]. Early symptoms include forefoot numbness and aching while late symptoms are mainly neuropathic dysesthesias. Diagnosis is primarily symptom based plus physical findings of either metatarsal head interspace tenderness or the Mulder click sign [4,6]. Interventional treatment consists of injections of local anesthetic mixed with either corticosteroids or 4% alcohol administered into the neuroma-containing interspace. If symptoms are severe and persistent or recurrent, neuroma excision is usually curative unless complicated by nerve regrowth and re-entrapment (stump neuroma).

When several of our patients reported both decreased RLS-type leg restlessness and improved quality of sleep after receipt of bilateral neuroma injections for neuropathic foot pains, their observations were considered potentially significant because of the known association of late onset RLS with neuropathic foot symptoms. This prompted a study of neuroma treatment in patients with RLS, including some without foot complaints. Of 15 patients with moderate to severe RLS, all of whom had bilateral physical findings of neuromas, treatment with injections or surgery resulted in sustained remission of RLS in nine with a concomitant marked improvement in subjective sleep quality and fatigue, indicating that their RLS was of peripheral (neuroma) origin [7]. In the current report, we describe a patient with severe, refractory RLS and PLMS with brain arousals (documented by polysomnography), the remission of which was prompt and near complete after bilateral excision of multiple foot neuromas.

## Case presentation

A 42-year-old Caucasian woman with a history of diabetes, depression, and human immunodeficiency virus (HIV) infection first had onset of bilateral foot tingling and numbness concomitant with an episode of acute severe pancreatitis. Over the next two years her foot dysesthesias progressed to burning discomfort, lancinating electric shock pains, and hypersensitivity. Examination by her foot specialist found physical signs indicative of bilateral Morton's neuromas, including third metatarsal head interspace tenderness and Mulder's clicks in each foot. She was given a series of neuroma injections (1 ml mixture of lidocaine, bupivacaine, methylprednisolone, dexamethasone and 4% alcohol). The injections improved the symptoms but relapses prompted bilateral third interspace neuroma excision ("initial neuroma surgery") which resulted in near-complete relief of neuropathic symptoms. After 6 months relatively mild foot dysesthesias recurred but resolved after the HIV drug stavudine was stopped.

**Figure 1:**
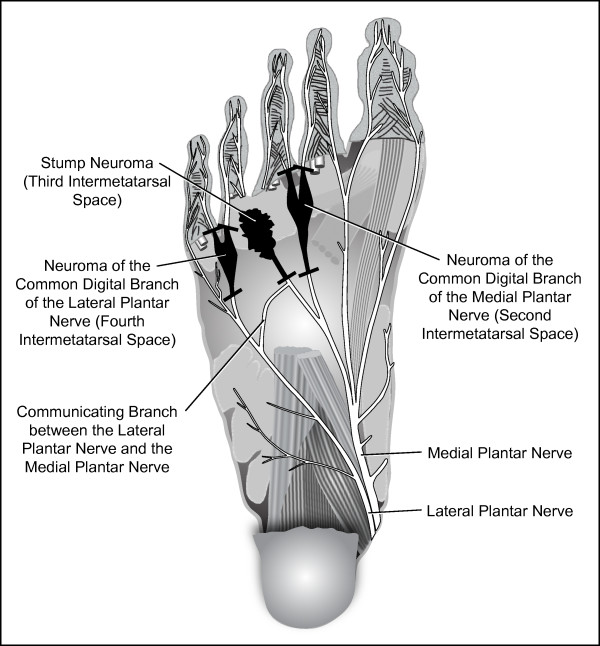
**Illustrative plantar view of the second, third, and fourth intermetatarsal space neuromas of the respective common digital branches of the medial and lateral plantar nerves of our patient's right foot (the left foot was essentially a mirror image)**. Short black bars indicate the points of nerve section for neuroma excision. The entrapping deep transverse metatarsal ligament lies dorsal to the neuromas and is not depicted.

Eighteen months after the initial neuroma surgery, fatigue, sleep difficulties, and sensorimotor symptoms typical of RLS were diagnosed in our patient. In retrospect, she had had RLS-like leg restlessness since childhood and she had noted that her leg restlessness had remitted for the 18 months since the initial neuroma surgery, which had been performed for neuropathic foot dysesthesias. Two third interspace stump neuroma injections improved leg restlessness, sleep quality and recurrent neuropathic dysesthesias. RLS and fatigue again worsened the following year but responded to pramipexole. Over the next two years she was maintained on pramipexole and intermittent neuroma injections, including several fourth interspace injections that also produced incremental improved sleep quality. However, leg restlessness, fatigue and fibromyalgia-like aches again relapsed severely and her recurrent foot dysesthesias required multiple daily doses of oxycodone-acetaminophen and tramadol. Polysomnography performed at that time showed severe PLMS and associated arousals (Table 1). Her sleep specialist then increased her pramipexole dosing and prescribed oral iron for low ferritin. Over the next two months our patient remained severely symptomatic, and concern for augmentation prompted cessation of pramipexole. Additional neuroma injections were not helpful. Over the next several months she was maintained on iron, gabapentin, tramadol, oxycodone-acetaminophen and duloxetine, but continued to be severely symptomatic with respect to fatigue, leg restlessness, sleep difficulties, and bilateral foot dysesthesias. An ultrasound scan of her feet at that time (now 5 years out from the initial neuroma surgery) showed third nerve stump neuromas and bilateral neuromas of the second and fourth digital nerves. Excision of all neuromas was recommended and informed consent was obtained. The second and fourth interspace neuromas and third interspace stump neuromas (Figure 1) were then excised ("second neuroma surgery") as previously described [8].

**Table 1:** Pre/post second neuroma surgery polysomnography results

	Pre-operative (Baseline) (-16 weeks)	Post-operative (+6.3 weeks)
Total sleep time (efficiency)	309 minutes (71%)	346 minutes (88%)

SLEEP STAGE%		

Stage 1	21.5%	03.3%

Stage 2	73.2%	64.0%

Stage 3/4	0%	0%

REM sleep	05.3%	32.7%

Respiratory events (AHI rate)	16 (3.1 events/hour)	47 (8.2 events/hour)

Periodic limb movements	782	61

Periodic limb movements with		

arousal (rate)	698 (135.5 movements/hour)	19 (3.3 movements/hour)

Spontaneous arousals (rate)	89 (17.3 arousals/hour)	0 (0 arousals/hour)

AHI = apnea hyperpnea index.

Severity of evening leg restlessness, daytime fatigue, overall quality of sleep, and neuropathic forefoot numbness, burning and/or tingling, electric shock pains, and foot hypersensitivity were separately assessed with 10 cm visual analog scales, with the zero score representing no symptoms and the 10 cm score the worst imaginable severity. RLS was scored by the International RLS Rating Scale (scoring range 0 to 40 points) [9]. Assessments of depressive symptomatology were performed using the Beck Depression Inventory II (scoring range 0 to 63 points, above 29 equals severe), sleep by the Pittsburgh Sleep Quality Index (scoring range 0 to 21 points, poor sleep is five or greater), and fatigue by the Fatigue Severity Scale (scoring range nine to 63 points, significant fatigue is 36 or greater), and the Multidimensional Assessment of Fatigue (scoring range 1 to 50 points, two or greater equals increasing level of fatigue). The baseline polysomnographic study was repeated six weeks after the second neuroma surgery. Each sleep study included bilateral electro-oculography, sub-mental electromyography, bilateral anterior tibialis electromyography, central and occipital electroencephalography, electrocardiographic waveform, airflow and respiratory effort assessment, oximetry, and video monitoring.

The pre-operative questionnaire and symptom scale assessments of leg restlessness, fatigue, neuropathic foot symptoms, sleep quality, and depression are shown in Table 2. All excised neuromas were confirmed histopathologically. Despite wound pains, our patient noted marked subjective improvement in sleep quality starting from the night following surgery. She began having nightly dreams. Sustained resolution of leg restlessness and neuropathic foot dysesthesias also occurred immediately following the neuroma surgery, and she remained completely off neuropsychiatric medication as well as iron and all other drugs known to affect RLS for the next six weeks. Post-operative questionnaire and symptom assessments were performed at 18 days and polysomnography was performed at six weeks (Tables 1 and 2). At seven weeks post-operatively, she noted bilateral leg edema that was unrelated to her foot surgery, and this was associated with recurrent mild fatigue as well as some hypersensitivity and burning discomfort of her feet bothersome enough to require occasional tramadol or oxycodone in the daytime and regular gabapentin at bedtime. Leg restlessness remained in remission and good sleep quality was maintained, as reflected in her nine-week follow-up questionnaire and symptom scale responses.

**Table 2:** Pre/post-second neuroma surgery questionnaire and rating scale results

	Pre-operative (baseline)	Post-operative		
	**(-1 week)**	**(+2.6 weeks)**	**(+9 weeks)**	**(+115 weeks)**

NEUROPATHIC SYMPTOM SCORES				

VAS forefoot numbness	0.5	0	0	7.6

VAS burning and/or tingling	7.9	0	2.5	4.1

VAS electric shock pains	8.8	1.4	1	3.8

VAS foot hypersensitivity	6.4	0	5.1	7.5

RLS SCORES				

International RLS rating scale	36	4	1	10

VAS evening leg restlessness	9.0	0.6	0	1.9

DEPRESSION SCORE				

Beck depression inventory II	29	1	1	8

FATIGUE SCORES				

VAS daytime fatigue	6.3	0	1.5	2.8

Fatigue severity scale	52.2	16.2	12.6	41

MAF Global fatigue index	35.1	1	12	15.1

SLEEP QUALITY SCORES				

VAS poor quality sleep	10.0	1	0	1.9

PSQI Global score	18	1	2	8

MAF = multidimensional assessment of fatigue; PSQI = Pittsburgh Sleep Quality Index; RLS = restless legs syndrome; VAS = visual analog scale.

By six months after the second neuroma surgery, her neuropathic dysesthesias had recurred to the point of requiring daily pregabalin dosing along with tramadol or oxycodone-acetaminophen. At six months her primary care physician also restarted citalopram for depression. At two years after the second neuroma surgery, her VAS scores for numbness and hypersensitivity exceeded the pre-operative baseline but scores for burning and/or tingling and lancinating electric shock pains remained about 50% or less of her baseline. Also after two years her RLS scores remained in the mild range and her scores for poor quality sleep and fatigue also remained much better than the baseline scores (Table 1). She remained off any RLS treatment except for the RLS benefiting effects of pregabalin and intermittent oxycodone or tramadol taken for foot dysesthesias.

## Discussion

Our patient's neuropathic foot dysesthesias first fully remitted after her initial neuroma surgery and again 5 years later after the second neuroma surgery. The first mild recurrence of dysesthesias resolved with stoppage of stavudine, a nucleoside HIV drug associated with neuropathic foot symptoms. Her HIV was fully suppressed with normal immune function throughout 10 years of treatment and was not considered to have any role in her ongoing symptoms otherwise. The remissions related to the two neuroma surgeries as well as the repeated improvements with neuroma injections indicate that her neuropathic foot dysesthesias were due to neuromas rather than small fiber neuropathy, which has been associated with diabetes and HIV infection.

Our patient clearly had RLS. Her symptoms fulfilled all the cardinal criteria for the diagnosis and her score of 36 on the 40-point International RLS rating scale was in the very severe range. The baseline polysomnogram showed a severe degree of PLMS and arousals. Over the next 4 months, despite a new regimen of drugs and additional neuroma injections, her foot dysesthesias, leg restlessness, non-restorative sleep, and fatigue all remained severe as shown by her pre-operative questionnaire and rating scale responses.

Surgery was recommended because of the severity and refractory nature of her symptoms. Excision of multiple neuromas was planned because (a) office ultrasound [10] documented large neuromas in the second and fourth metatarsal head interspaces in addition to stump neuromas of the third interspaces, (b) physical findings were present (Mulder's clicks in the third and fourth interspaces and tenderness in all three interspaces bilaterally), and (c) our (unpublished) neuroma injection experience has been that second and fourth interspace neuromas may contribute to neuropathic symptoms and sleep dysfunction, respectively.

Post-operatively her clinical improvement with respect to RLS symptoms, subjective sleep quality and neuropathic foot dysesthesias was immediate, and near total. That all symptoms originated peripherally from neuromas is supported by the histopathological documentation of the excised tissues and the fact she remained clinically well completely off neuropsychiatric medication for the initial six weeks after surgery. The follow-up polysomnography is notable for improved sleep efficiency and a substantial increase in REM sleep. It also documented both a marked reduction in arousals associated with PLMS and an apparent elimination of spontaneous arousals. The rapid reduction of her fatigue scores suggests that most of her fatigue was due to poor sleep quality.

We have previously proposed that the leg symptoms of RLS, and possibly also PLMS and arousals, may be due to afferent nerve impulses generated from the entrapment and compression-related digital nerve irritability and damage associated with foot neuromas [9], and this hypothesis is supported by the clinical and polysomnographic results from our patient. In our cumulative experience with over 100 patients with RLS, we have determined that they uniformly have bilateral foot signs or ultrasound evidence of neuromas irrespective of whether they have foot sensory symptoms or not and regardless of whether their RLS is primary or secondary, or is of early or late onset. Third interspace neuromas would likely be the major source of RLS because bilateral injections of this interspace alone can induce remission of RLS symptoms [9]. The possible contribution to neuropathic foot symptoms and sleep dysfunction of the second and fourth interspace neuromas remains to be better delineated. That some PLMS still occurred post-operatively may reflect afferent impulses arising from her freshly cut digital nerve stumps. The eventual recurrence of mild leg restlessness and the relapse of neuropathic foot symptoms in our patient starting at seven weeks post-neuroma removal may indicate renewed nerve stump entrapment(s) as a result of digital nerve regrowth and scarring. This occurrence is the unpredictable downside of neuroma excision and it underscores the reality that current techniques for neuroma resection are not necessarily curative. However, although PLMS and arousals had likely also relapsed to some degree by two years out from the second neuroma surgery, her scores for sleep quality and fatigue remained much better than her pre-operative baseline and she has never regretted that she had had the multiple neuromas excised.

## Conclusions

The immediate and near-complete remission of symptoms, the histopathological documentation, and the marked improvement in polysomnographic parameters together clearly indicate that our patient's severe RLS and PLMS was of peripheral nerve (foot neuroma) origin. Further study of foot neuromas as a source of PLMS and as a cause of sleep dysfunction in patients with or without concomitant RLS, is warranted.

## Consent

Written informed consent was obtained from the patient for publication of this case report and any accompanying images. A copy of the written consent is available for review by the journal's Editor-in-Chief.

## Competing interests

The authors declare that they have no competing interests.

## Authors' contributions

LAL directed our patient's care, administered and interpreted the rating scales and questionnaires, and wrote the manuscript. CJG administered all neuroma injections, performed and interpreted all ultrasound evaluations, performed the neuroma surgeries, and contributed to the methods portion of the manuscript. TDK helped manage our patient's RLS and performed and interpreted the polysomnographic studies. All authors read and approved the final manuscript.
